# Supplementary material for: Quantifying Metagenomic Strain Associations from Microbiomes with Anpan
Source: bioRxiv. 2025 Jan 7:2025.01.06.631550. Preprint. [Version 1] doi: 10.1101/2025.01.06.631550 (PMC11741421; doi:10.1101/2025.01.06.631550)
Supplement: Supplement 1 — Figure S1: Adaptive filtering of simulated data shows accurate, conservative labeling of samples. The k-means filter applied to simulated data, alongside the true simulated status of each sample. While there are false negatives included in this example, there are no false positives as well, indicating that for these simulation parameters, the filter errs on the side of being conservative. [file media-1.pdf]

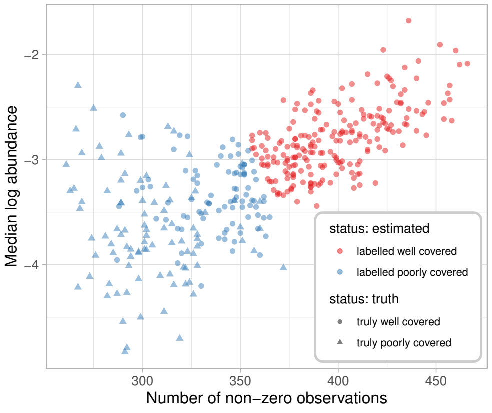
Median log abundance
−2
−3
−4
300
350
400
450
Number of non−zero observations
status: estimated
labelled well covered
labelled poorly covered
status: truth
truly well covered
truly poorly covered
