# Supplementary figures and images for: Quantifying Metagenomic Strain Associations from Microbiomes with Anpan
Source: bioRxiv. 2025 Jan 7:2025.01.06.631550. Preprint. [Version 1] doi: 10.1101/2025.01.06.631550 (PMC11741421; doi:10.1101/2025.01.06.631550)

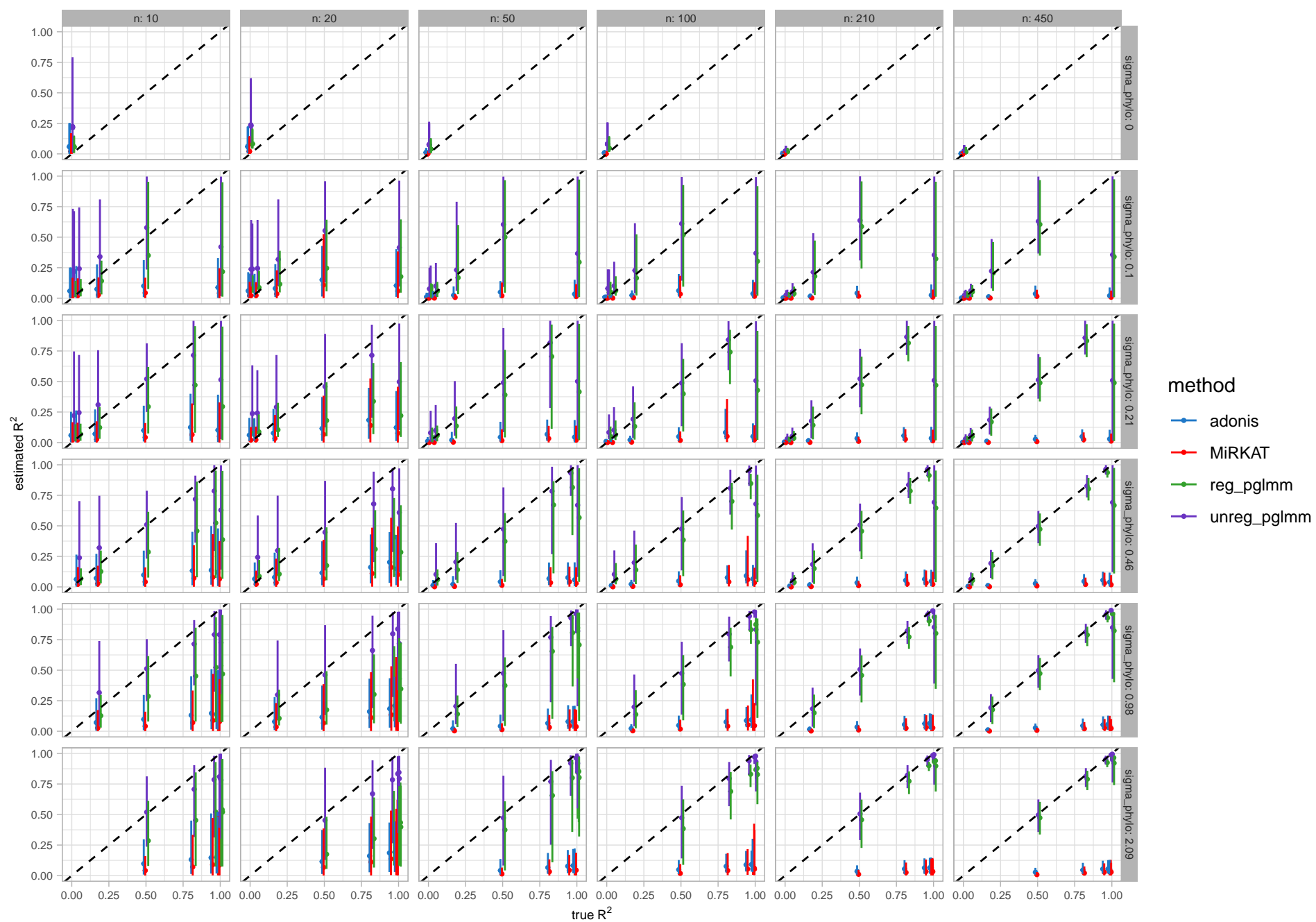

Supplement: Supplement 2 — Figure S2: Simulated R2 estimates with varying σP. Extended results from Fig. 4A. Permutational linear models consistently underestimate R2 across all values of σP. [file media-2.pdf]

Clostridium\_bolteae (n = 268)  
25 genes with Q below 0.1 and abs(coefficient) above 1

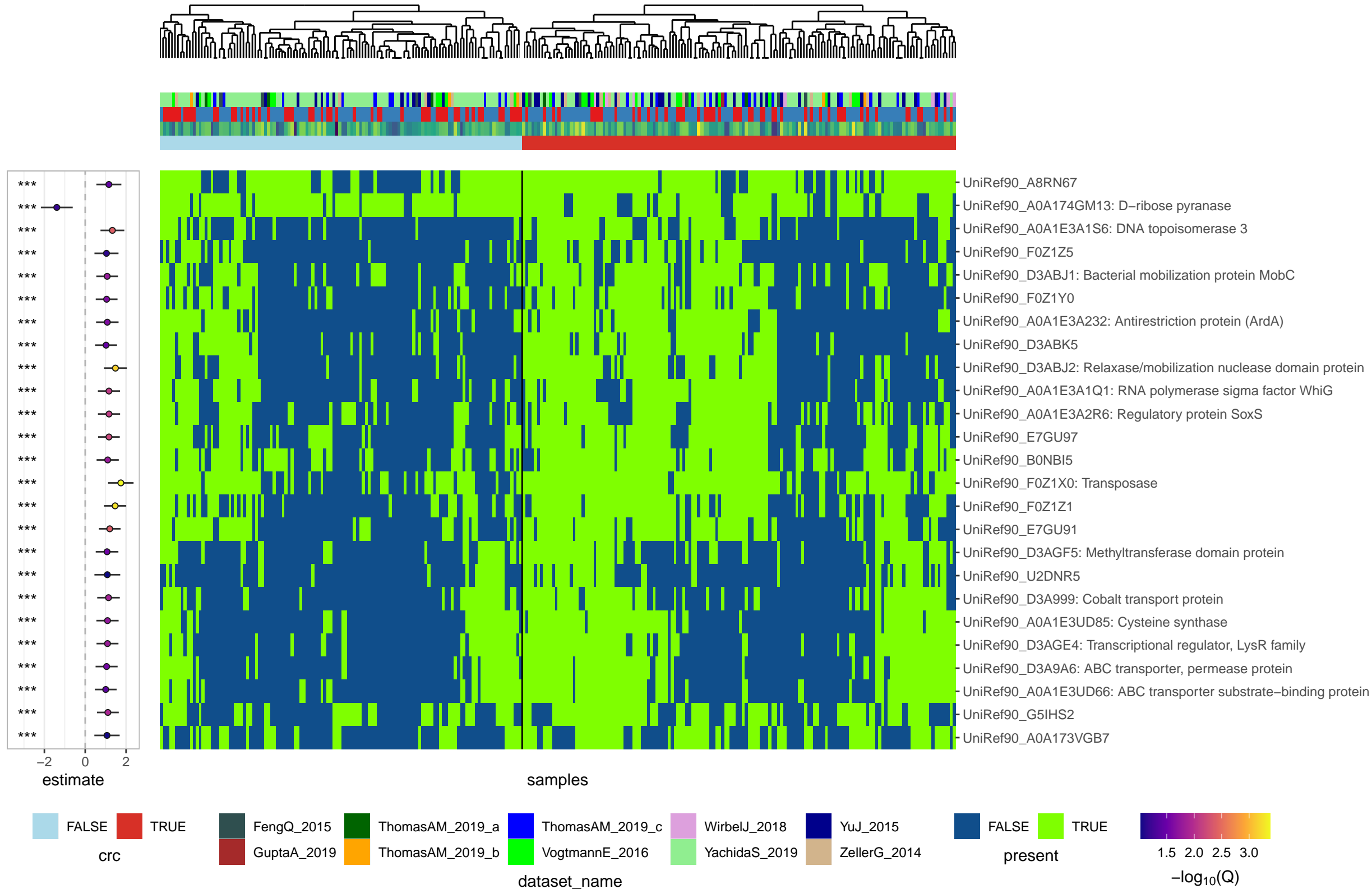

Supplement: Supplement 3 — Figure S3: Heatmap results plot of genes in C. bolteae. The heatmap shows 25 genes in C. bolteae falling withing thresholds Q < .01 and with absolute coefficients greater than 1. Highlighted is UniRef90_A0A1E3A1S6, a topoisomerase more prevalent in CRC cases. This result aligns with the hypothesis that genes enabling genomic restructuring become more prevalent in CRC-derived samples by encouraging adaptation to the inflammatory environment present in the CRC gut. [file media-3.pdf]
